# Genome-wide characterization and expression profiling of *SWEET* genes in cabbage (*Brassica oleracea var. capitata* L.) reveal their roles in chilling and clubroot disease responses

**DOI:** 10.1186/s12864-019-5454-2

**Published:** 2019-01-29

**Authors:** Wei Zhang, Shenyun Wang, Fangwei Yu, Jun Tang, Xi Shan, Kan Bao, Li Yu, Hong Wang, Zhangjun Fei, Jianbin Li

**Affiliations:** 1 Jiangsu Key Laboratory for Horticultural Crop Genetic Improvement, Institute of Vegetable Crops, Jiangsu Academy of Agricultural Sciences, Nanjing, 210014 People’s Republic of China; 2 Zhenjiang Agricultural Research Institute, Jurong, Jiangsu 212400 People’s Republic of China; 3 Boyce Thompson Institute, Cornell University, Ithaca, NY 14853 USA

**Keywords:** Cabbage (*Brassica oleracea var. capitata* L.), Expression profile, RNA-Seq, Stress response, SWEET

## Abstract

**Background:**

The SWEET proteins are a group of sugar transporters that play a role in sugar efflux during a range of biological processes, including stress responses. However, there has been no comprehensive analysis of the *SWEET* family genes in *Brassica oleracea* (*BoSWEET*), and the evolutionary pattern, phylogenetic relationship, gene characteristics of *BoSWEET* genes and their expression patterns under biotic and abiotic stresses remain largely unexplored.

**Results:**

A total of 30 *BoSWEET* genes were identified and divided into four clades in *B. oleracea*. Phylogenetic analysis of the BoSWEET proteins indicated that clade II formed first, followed by clade I, clade IV and clade III, successively. Clade III, the newest clade, shows signs of rapid expansion. The *Ks* values of the orthologous *SWEET* gene pairs between *B. oleracea* and *Arabidopsis thaliana* ranged from 0.30 to 0.45, which estimated that *B. oleracea* diverged from *A. thaliana* approximately 10 to 15 million years ago. Prediction of transmembrane regions showed that eight BoSWEET proteins contain one characteristic MtN3_slv domain, twenty-one contain two, and one has four. Quantitative reverse transcription-PCR (qRT-PCR) analysis revealed that five *BoSWEET* genes from clades III and IV exhibited reduced expression levels under chilling stress. Additionally, the expression levels of six *BoSWEET* genes were up-regulated in roots of a clubroot-susceptible cabbage cultivar (CS-JF1) at 7 days after inoculation with *Plasmodiophora brassicae* compared with uninoculated plants, indicating that these genes may play important roles in transporting sugars into sink roots associated with *P. brassicae* colonization in CS-JF1. Subcellular localization analysis of a subset of BoSWEET proteins indicated that they are localized in the plasma membrane.

**Conclusions:**

This study provides important insights into the evolution of the *SWEET* gene family in *B. oleracea* and other species, and represents the first study to characterize phylogenetic relationship, gene structures and expression patterns of the *BoSWEET* genes. These findings provide new insights into the complex transcriptional regulation of *BoSWEET* genes, as well as potential candidate *BoSWEET* genes that promote sugar transport to enhance chilling tolerance and clubroot disease resistance in cabbage.

**Electronic supplementary material:**

The online version of this article (10.1186/s12864-019-5454-2) contains supplementary material, which is available to authorized users.

## Background

Sugars as essential energy sources, are synthesized in leaves (source organs) and then translocated via phloem sap into sink organs, such as modified leaves, roots, seeds and fruits, and the amount of sugars influences plant development [1, 2]. In plants, sugar transport is mediated by proteins in the sucrose transporter (SUT) and monosaccharide transporter (MST) and SWEET families [3, 4]. SWEET, a novel class of sugar transporters, is a distinct transporter family which mediates influx or efflux of sugars from phloem parenchyma into the phloem apoplast [5–7]. In prokaryotes SWEET proteins contain only three transmembrane helices (TMHs), while in eukaryotes there are also examples with seven TMHs. The seven-TMHs has evolved with two tandemly repeated three-TMH units separated by a single TMH [8]. Phylogenetic analysis of SWEET proteins has shown that they can be divided into four clades [3], with clades I and II preferentially transporting hexoses, clade III transporting sucrose, and clade IV being responsible for the flux of fructose across the tonoplast [9–12].

With the rapid development of whole-genome sequencing, genome-wide identification of *SWEET* genes in various species have been reported, such as in *Arabidopsis thaliana* [3], rice (*Oryza sativa*) [13], sorghum (*Sorghum bicolor*) [14], soybean (*Glycine max*) [15], apple (*Malus domestica*) [16], grape (*Vitis vinifera*) [17], banana (*Musa acuminate*) [18], tomato (*Solanum lycopersicum*) [19] and rapeseed (*Brassica napus*) [20]. Biochemical and functional analyses have shown that *SWEET* genes play significant roles in various physiological processes, such as nectar secretion [21, 22], seed and pollen development [23, 24], leaf senescence [25], and responses to abiotic [12, 26–28] and biotic stresses [3, 17, 29–31]. For example, *AtSWEET8* is essential for pollen viability in *A. thaliana*, and decreased expression reduces starch content in pollen grains and causes male sterility [3]. Moreover, the *SWEET* genes are critical for carbon transport regulation in host-pathogen interactions and have been shown to be targets of extracellular pathogens [30]. Induction of *SWEET* genes upon pathogen infection has also been reported in alfalfa (*Medicago sativa*), rice, grape, *A. thaliana* and Chinese cabbage (*Brassica rapa*) [29–34]. For example, *MtN3*, the first identified member of the *SWEET* family, was found to participate in the host-*Rhizobium meliloti* interaction in alfalfa [32]. *OsSWEET11*, *OsSWEET13* and *OsSWEET14* were later shown to be associated with resistance to bacterial blight in rice [29, 31, 33]. In grape, the expression of *VvSWEET4* increased after *Botrytis cinerea* infection [17].

Clubroot disease is a soil-borne disease caused by the obligate biotrophic pathogen *P. brassicae* and is one of the most devastating diseases in Brassicaceae plants, reducing both crop quality and yields [35, 36]. The life cycle of *P. brassicae* consists of three stages: the survival stage of resting spores in the soil, the primary infection (root hair infection) stage and the secondary infection (root cortex infection) stage [37–39]. The resting spores can survive in the soil for 6–12 years, making this disease hard to control once the soil has been contaminated [40]. In *A. thaliana* after *P. brassicae* infection the expression of *AtSWEET15* was strongly induced during gall formation, and the *atsweet11* mutant exhibited slower gall formation compared to wild-type plants [30, 34]. In Chinese cabbage, the expression of several *BrSWEET* genes from Clade I and III increased, as did glucose and fructose levels, in roots of a clubroot-susceptible line compared to a clubroot-resistant line following *P. brassicae* infection, suggesting a close relationship between *P. brassicae* growth, sugar translocation and the expression of *BrSWEET* genes [30].

Cold stress (CS), including chilling (< 20 °C) and freezing (< 0 °C), has a major impact on plant growth and development, limiting geographic distribution and productivity [41]. It has been long established that accumulation of soluble sugars can stabilize cellular components and membranes following CS [42, 43]. Overexpression of *AtSWEET4* has been shown to increase plant size and freezing tolerance in *A. thaliana* [28], while cold-stressed *AtSWEET16* overexpressing lines are unable to accumulate fructose and have increased tolerance to freezing stress [26]. The fructose-specific transporter AtSWEET17 plays a primary role in fructose homeostasis following 1 week of 4 °C treatment [12]. Interestingly, the double mutant *atsweet11*/*atsweet12* was reported to release fewer electrolytes when the temperature was reduced to 4 °C, but exhibited greater freezing tolerance than both single mutants and wild-type *A. thaliana* [27].

Cabbage (*Brassica oleracea var. capitata* L.), belonging to the Brassicaceae family, is one of the most economically important leafy vegetable crops worldwide. Chilling and clubroot disease cause severe losses of yields and quality in this species, as well as in other Brassicaceae crops. Even though they have been associated with responses to chilling and clubroot disease in other species, little is known about the role of the SWEET sugar transporters in chilling and clubroot disease responses in cabbage. The objectives of this study were to conduct a genome-wide analysis of the *SWEET* gene family in *B. oleracea* and thirteen other species, and to develop a better understanding of the molecular evolution and function of the SWEET proteins in cabbage, while also providing a reference for other Brassicaceae species.

## Methods

### Identification of *SWEET* family genes in *B. oleracea* and thirteen other plant species

The *B. oleracea* whole-genome sequence used to identify the *BoSWEET* genes was downloaded from the *B. oleracea* Genome Database (Bolbase, http://ocri-genomics.org/bolbase/) [44]. The amino acid sequences of the *A. thaliana SWEET* genes were retrieved from the TAIR database (http://www.arabidopsis.org/). The *Brassica rapa* whole-genome sequence was obtained from the BRAD database (http://brassicadb.org/brad/) [45]. The *Carica papaya*, *Populus trichocarpa*, *Vitis vinifera*, *Oryza sativa*, *Zea mays*, *Selaginella moellendorffii*, *Physcomitrella patens, Chlamydomonas reinhardtii*, *Volvox carteri*, *Ostreococcus lucimarinus* and *Ostreococcus tauri* gene information was downloaded from the Plaza v2.5 database (http://bioinformatics.psb.ugent.be/plaza/news/index) [46]. The Hidden Markov Model (HMM) corresponding to the MtN3/saliva (MtN3_slv) domain (PF03083) was retrieved from Pfam 31.0 (http://pfam.xfam.org/) and used to identify putative SWEET proteins with the “trusted cutoff” as the threshold [47, 48]. AtSWEET protein sequences were used as the seed sequences to carry out a BLASTP search in the sequences from the other species with an *E*-value threshold of 1e^− 10^. The Pfam 31.0 database, the SMART database (http://smart.embl-heidelberg.de/) and the Conserved Domain Database (http://www.ncbi.nlm.nih.gov/Structure/cdd/wrpsb.cgi/) were then used to further filter and analyze the potential SWEET protein sequences to validate the HMM and BLASTP search results [49, 50]. The SWEET protein sequences from *B. oleracea* and *B. rapa* were named by adding a suffix (a, b, c...etc.) based on sequence similarity to the corresponding AtSWEET proteins.

### Phylogenetic analysis and characterization of BoSWEET proteins

The ProtParam tool (https://web.expasy.org/protparam/) was used to analyze the physical and chemical parameters of the BoSWEET proteins, including molecular weight and theoretical pI. The Gene Structure Display Server (GSDS, http://gsds.cbi.pku.edu.cn/index.php) was utilized to draw a schematic diagram of the gene structure according to the genomic sequences and the corresponding coding sequence of each *BoSWEET* gene [51]. The online MEME tool (http://meme-suite.org/tools/meme) was used to identify conserved protein motifs using default parameters [52]. The TMHs of BoSWEET proteins were predicted by the TMHMM Server v.2.0 (http://www.cbs.dtu.dk/services/TMHMM/). The Conserved Domain Architecture Retrieval Tool (CDART) (http://www.ncbi.nlm.nih.gov/Structure/lexington/lexington.cgi) was used to draw the MtN3_slv domains [53]. The SignalP 4.1 server (http://www.cbs.dtu.dk/services/SignalP/) was used to predict the presence and location of signal peptide cleavage sites in the amino acid sequences. Multiple alignment of all the BoSWEET proteins was performed using ClustalW [54], and the phylogenetic tree was constructed by MEGA7 with the bootstrap of 1000 replicates using the neighbor-joining (NJ) method [55].

### Chromosomal localization of *BoSWEET* genes, and identification of orthologs and paralogs

The chromosomal localization of the *BoSWEET* genes was determined using MapChart 2.30 [56]. OrthoMCL (http://orthomcl.org/orthomcl/) was used to identify the orthologs and paralogs of the SWEET proteins in *B. oleracea* and *A. thaliana*. The relationships of orthologs and paralogs were plotted using the Circos software [57].

The occurrence of duplication events and divergence time of orthologous genes, as well as the selective pressure on duplicated genes, was estimated by calculating *Ks* (synonymous substitution rate) and *Ka* (nonsynonymous substitution rate) values using DnaSP 6 [58]. The *Ks* values of all the syntenic orthologs of the *SWEET* genes between *B. oleracea* and *A. thaliana* were then plotted as the density using an R package [59]. The divergence time was calculated using the formula, T = *Ks*/2r, with the value of r being 1.5 × 10^− 8^ synonymous substitutions per site per year for dicotyledonous plants [60].

### Chilling stress, sample collection and quantitative reverse transcription-PCR (qRT-PCR)

To investigate *BoSWEET* expression profiles in response to chilling stress, the advanced inbred cold-tolerance cabbage line 923 (CT-923) was used. Seeds were grown in sterilized soil in a growth chamber at 25 °C day /18 °C night, with a photoperiod of 14 h light/10 h dark. After 4 weeks, to induce chilling stress, the seedlings were maintained at 4 °C for 0, 3, 6, 12, 24 and 48 h. Next, samples were collected and immediately frozen in liquid nitrogen and stored at − 80 °C till RNA extraction. For quantitative gene expression analysis, total RNA was extracted using the TaKaRa MiniBEST Plant RNA Extraction Kit according to the manufacturer’s instructions (Takara Bio Inc., Dalian, China). The first-strand cDNA was synthesized using the PrimeScript™ RT reagent Kit with gDNA Eraser (TakaRa). The qRT-PCR reactions were performed using TB Green™ Premix Ex Taq™ II (TakaRa) and carried out on a Roche LightCycler® 480II PCR System. Gene specific primers were designed using Beacon Designer 7.7 (Premier Biosoft, CA, USA) and are listed in Additional file 1: Table S1. *BoActin2* was used as the reference gene [61]. All reactions were performed in triplicate, and the 2^-△△CT^ method was applied to calculate the relative expression [62]. Duncan’s multiple range test at *P* < 0.05 was used to determine the significance level of the data, using the SPSS 21 software (SPSS Inc., USA).

### RNA-Seq data analysis of *BoSWEET* genes

To analyze the *BoSWEET* expression profiles in different organs and in response to *P. brassicae* infection, RNA-Seq data from the GSE42891 (GEO database) and PRJNA453960 (BioProject accession) were downloaded from NCBI. The GSE42891 RNA-Seq data contained expression profiles of seven different organs/tissues (bud, callus, flower, leaf, root, silique and stem) of the cabbage homozygous line 02–12 [44]. The PRJNA453960 RNA-Seq data contained the root expression profiles from clubroot-resistant cabbage Xiangan336 (CR-XG336) and clubroot-susceptible cabbage Jingfeng No.1 (CS-JF1) at 7 (primary infection stage) and 28 (clubroot formation stage) days after inoculation (DAI) with *P. brassicae*. The corresponding root samples at 7 d and 28 d without inoculation were sampled as the mock control, and all the samples were collected in three biological replicates. Raw RNA-Seq reads were processed to trim the adapter and low-quality sequences using Trimmomatic [63]. The high-quality cleaned reads were aligned to the *B. oleracea* genome using HISAT [64] allowing up to 3 edit distances. Following alignments, raw counts for each gene were derived and normalized into FPKM (fragments per kilobase of exon model per million mapped reads). Raw count data was then fed to DESeq2 [65] to identify differentially expressed genes with a cut off of fold change > 2 and FDR < 0.05. Heatmap of *BoSWEET* gene expression profiles was generated using the pheatmap package (https://cran.r-project.org/web/packages/pheatmap/) based on the log_2_ transformed FPKM values.

### Construction of BoSWEET transient expression vectors and subcellular localization studies in tobacco

To investigate the subcellular localization of the BoSWEET proteins, they were transiently expressed as translational GFP (green fluorescent protein) fusion proteins in tobacco (*Nicotiana benthamiana*) leaf epidermal cells. The full-length coding sequences of *BoSWEET11b*, *BoSWEET11c* and *BoSWEET12b* were amplified using a forward primer containing a *Kpn* I restriction site and a reverse primer containing a *Xba* I restriction site. The primers used are listed in Additional file 1: Table S1. The amplification products were digested with *Kpn* I and *Xba* I and ligated into the pCAMBIA1300-35S-GFP (35S-GFP) vector. The recombined plasmids were then transformed into *Agrobacterium tumefaciens* strain GV3101 [66]. *Agrobacterium* transient expression and infiltration was carried out according to previously published protocols [67]. Leaves transformed with the 35S-GFP vector alone were used as controls. Two days after infiltration, fluorescence and bright-light images of transiently infected tobacco leaves were obtained using a fluorescence microscope (BX41, Olympus, Rungis, France).

## Results

### Evolutionary history of *SWEET* genes among plant species

**Fig. 1:**
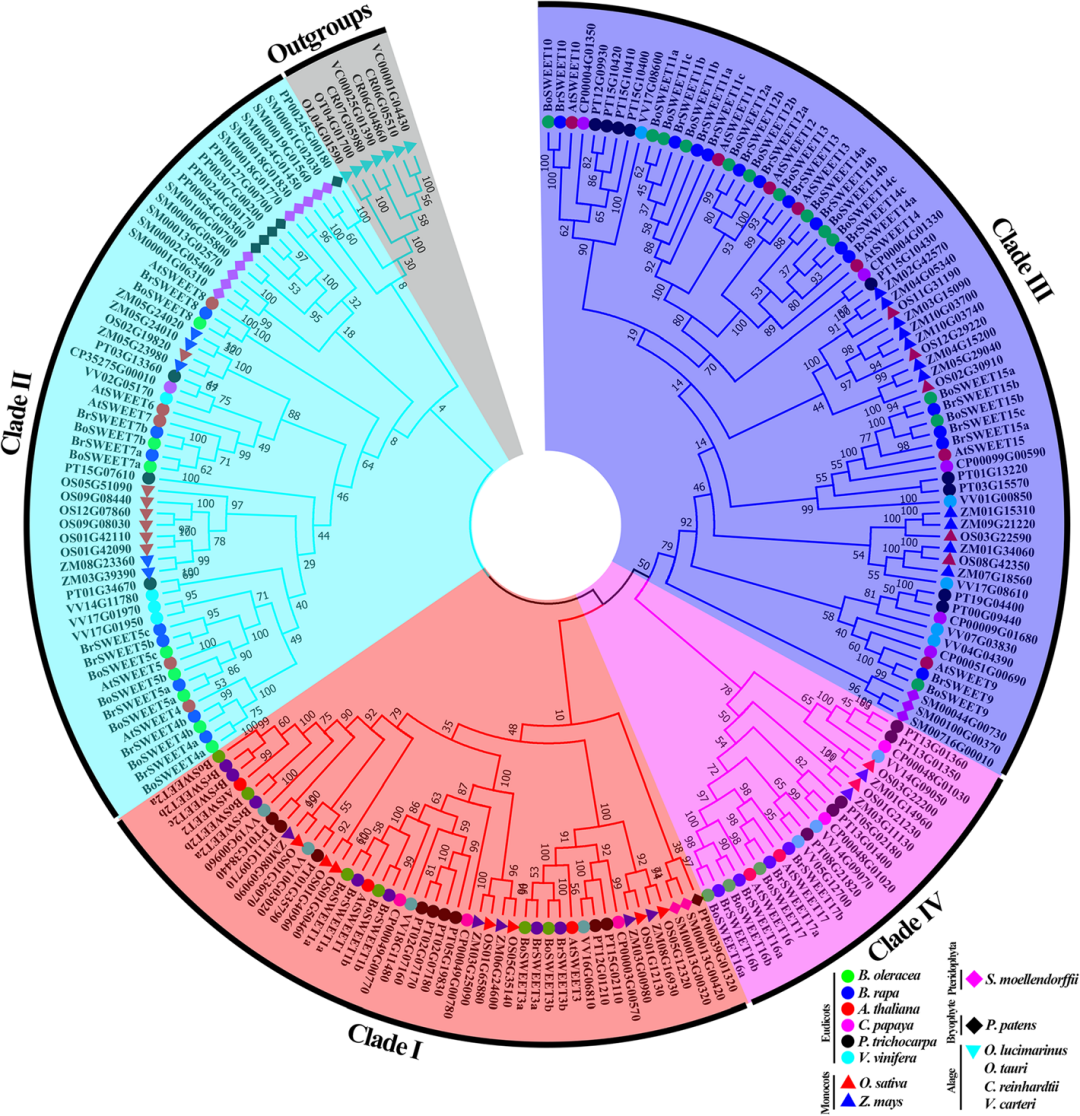
Phylogenetic relationships among *SWEET* genes in 14 plant species. The species abbreviations are as follows: *Brassica oleracea* (Bo), *Brassica rapa* (Br), *Arabidopsis thaliana* (At), *Carica papaya* (Cp), *Populus trichocarpa* (Pt), *Vitis vinifera* (Vv), *Oryza sativa* (Os), *Zea mays* (Zm), *Selaginella moellendorffii* (Sm*)*, *Ostreococcus lucimarinus* (Ol), *Ostreococcus tauri* (Ot), *Chlamydomonas reinhardtii* (Cr), *Volvox carteri* (Vc). The phylogenetic tree was constructed using the neighbor-joining (NJ) method and with 1000 bootstrap replications. The numbers at the nodes represent bootstrap percentage values. Genes from each species are marked with different colors/shapes. Clades I, II, IV, and III are indicated by red, cyan, blue and carmine, respectively. The *SWEET* genes from *O. lucimarinus*, *O. tauri*, *C. reinhardtii*, and *V. carteri* were selected as the outgroups

All putative *SWEET* genes were identified in *B. oleracea* and thirteen other representative plant species (Additional file 2: Table S2). A phylogenetic tree of the identified 205 *SWEET* genes from the 14 species was constructed in order to investigate the evolutionary history of the family in the plant kingdom, with genes from the four algal species selected as the outgroups (Fig. 1, Additional file 2: Table S2). All other genes in the tree were clustered into four clades (clade I, clade II, clade III and clade IV) that were named according to the previously reported *A. thaliana* nomenclature [3]. According to the evolutionary distance with the outgroups, clade II formed first, followed by clade I, clade IV and clade III, with the latter expanding most rapidly (Fig. 1). The 30 *BoSWEET* and 33 *BrSWEET* genes identified in this study were named sequentially from *BoSWEET1* to *BoSWEET17* and *BrSWEET1* to *BrSWEET17*, respectively, according to their *A. thaliana* homologs.

**Fig. 2:**
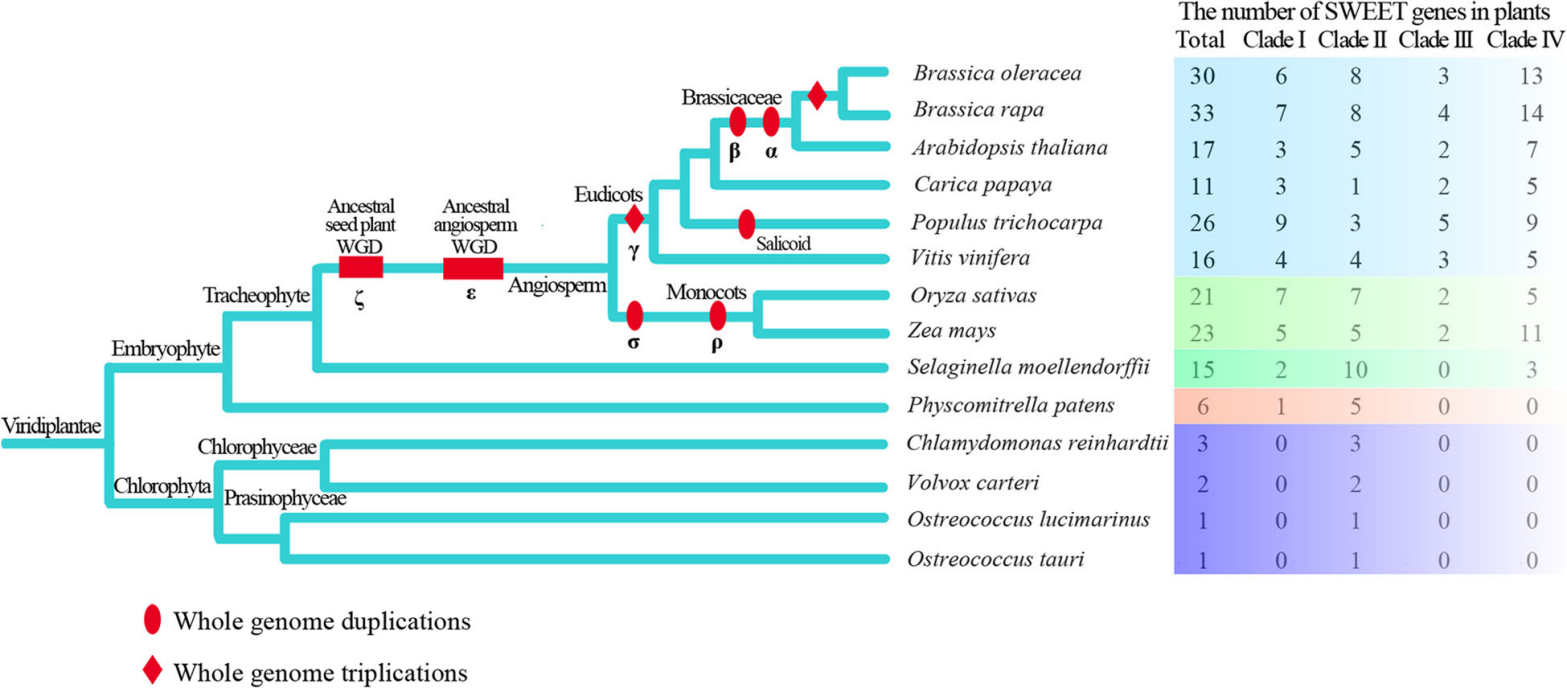
Number of *SWEET* genes from 14 plant genomes in four clades. Horizontal bars (ζ and ε) denote confidence regions for ancestral seed plant whole genome duplication (WGD) and ancestral angiosperm WGD; The γ and *Brassica*-specific triplication, α, β, σ, ρ and salicoid duplication are indicated on the branches of the tree according to previous reports [45, 83]

The phylogenetic relationships between the genes in the four clades were investigated (Fig. 2). In total of 14 plant species included dicotyledonous species (*B. oleracea*, *B. rapa*, *A. thaliana*, *Carica papaya*, *P. trichocarpa* and *V. vinifera*), monocotyledonous species (*O. sativa* and *Z. mays*), a pteridophyte (*S. moellendorffii*), a bryophyte (*P. patens*) and algae (*C. reinhardtii*, *V. carteri*, *O. lucimarinus* and *O. tauri*). In an evolutionary context, examples of genes in Clade II were first observed in the four algal species (unicellular chlorophyta), which contained fewer *SWEET* members (1–3), and clade I and clade IV were predominantly observed in *P. patens* (bryophyte) and *S. moellendorffii* (pteridophyta), respectively. Clade III was first formed in monocots. In eudicots, all six selected species underwent the γ triplication event, and the larger number of *SWEET* genes in *P. trichocarpa* than in *C. papaya* and *V. vinifera* is likely due to the salicoid-specific genome duplication [68]. As consequence of the α and β duplication events that occurred after the divergence of Brassicales, *A. thaliana* also has more members than *C. papaya*. Finally, the number of *SWEET* genes in *B. oleracea* and *B. rapa* apparently doubled after the *Brassica*-specific whole-genome triplication (WGT) event, based on a comparison with *A. thaliana* [45].

### Identification and phylogenetic analysis of *SWEET* genes in *B. oleracea*

We constructed an unrooted phylogenetic tree (Additional file 3: Figure S1) of SWEET proteins in *B. oleracea*, *B. rapa* and *A. thaliana*. A total of 80 members clustered into four clades, containing 16, 21, 34, and 9 members, respectively. Based on the tree, eleven duplication clusters in *B. oleracea* were identified and are listed in Table 1.

**Table 1:** *SWEET* family genes in *B. oleracea* and corresponding orthologs in *A. thaliana*

Group	tPCK Chr	CCB	*Arabidopsis thaliana*				*Brassica oleracea*								
			Gene name	Gene ID	ORF (bp)	PL (aa)	Gene name	Gene ID	Chromosome	Subgenome	ORF (bp)	PL (aa)	MW (kD)	pI	Number of MtN3_slv
Clade I	tPCK1	B	*AtSWEET1*	*AT1G21460*	744	247	*BoSWEET1a*	*Bol012680*	Scaffold000195_P1	MF1	741	246	27.805	9.2	2
							*BoSWEET1b*	*Bol009655*	C05	LF	756	251	27.111	9.38	2
	tPCK2	F	*AtSWEET2*	*AT3G14770*	711	236	*BoSWEET2a*	*Bol002799*	Scaffold000374	LF	711	236	26.683	8.95	2
							*BoSWEET2b*	*Bol011848*	Scaffold000206	MF1	594	197	22.330	8.58	2
	tPCK5	W	*AtSWEET3*	*AT5G53190*	792	263	*BoSWEET3a*	*Bol017165*	C07	MF1	780	259	29.239	8.6	2
							*BoSWEET3b*	*Bol030288*	C09	LF	648	215	24.056	7.64	1
Clade II	tPCK7	L	*AtSWEET4*	*AT3G28007*	756	251	*BoSWEET4a*	*Bol042944*	C07	LF	747	248	27.479	9.07	2
							*BoSWEET4b*	*Bol031203*	C02	MF1	738	245	27.323	8.78	2
	tPCK7	X	*AtSWEET5*	*AT5G62850*	723	240	*BoSWEET5a*	*Bol019179*	Scaffold000133	MF1	636	211	24.316	8.92	2
							*BoSWEET5b*	*Bol019426*	C06	LF	738	245	27.918	9.3	2
							*BoSWEET5c*	*Bol020702*	Scaffold000121_P2	MF2	1428	475	53.510	8.36	4
	tPCK6	E	*AtSWEET6*	*AT1G66770*	786	261	*/*	*/*	/	/	/	/	/	/	/
	tPCK5	P	*AtSWEET7*	*AT4G10850*	777	258	*BoSWEET7a*	*Bol006816*	C09	LF	750	249	27.277	9.56	2
							*BoSWEET7b*	*Bol025689*	C03	MF1	444	147	16.200	7.78	1
	tPCK4	S	*AtSWEET8*	*AT5G40260*	720	239	*BoSWEET8*	*Bol012888*	Scaffold000192	LF	717	238	26.670	8.9	2
Clade III	tPCK3	J	*AtSWEET9*	*AT2G39060*	777	258	*BoSWEET9*	*Bol020406*	C03	MF2	531	176	19.551	7.85	1
	tPCK5	W	*AtSWEET10*	*AT5G50790*	870	289	*BoSWEET10*	*Bol001620*	Scaffold000431	MF2	870	289	33.058	9.2	2
	tPCK6	M	*AtSWEET11*	*AT3G48740*	870	289	*BoSWEET11a*	*Bol003199*	Scaffold000361	MF1	534	177	19.181	8.68	1
							*BoSWEET11b*	*Bol037413*	C08	LF	870	289	31.945	9.32	2
							*BoSWEET11c*	*Bol002048*	Scaffold000407	MF2	534	177	19.208	8.98	1
	tPCK7	Q	*AtSWEET12*	*AT5G23660*	858	285	*BoSWEET12a*	*Bol036230*	C09	MF2	834	277	30.623	9.14	2
							*BoSWEET12b*	*Bol017126*	C07	LF	867	288	31.782	9.07	2
	tPCK5		*AtSWEET13*	*AT5G50800*	885	294	*BoSWEET13*	*Bol045233*	C06	/	600	199	22.122	8.49	1
	tPCK4	U	*AtSWEET14*	*AT4G25010*	846	281	*BoSWEET14a*	*Bol002776*	Scaffold000375	MF2	819	272	29.971	9.18	2
							*BoSWEET14b*	*Bol039481*	C01	LF	822	273	30.114	9.2	2
							*BoSWEET14c*	*Bol042202*	C07	MF1	612	203	22.429	9.12	1
	tPCK5	R	*AtSWEET15*	*AT5G13170*	879	292	*BoSWEET15a*	*Bol034222*	C03	MF2	705	234	26.232	6.58	2
							*BoSWEET15b*	*Bol043455*	C09	LF	540	179	19.812	6.28	1
Clade IV	tPCK2	F	*AtSWEET16*	*AT3G16690*	693	230	*BoSWEET16a*	*Bol034791*	C01	MF1	696	231	25.759	8.69	2
							*BoSWEET16b*	*Bol022976*	C03	MF2	552	183	20.362	9.35	2
	tPCK4	T	*AtSWEET17*	*AT4G15920*	726	241	*BoSWEET17*	*Bol037218*	C07	MF2	723	240	26.513	6.73	2

*Note*: *tPCK Chr* Chromosome of translocation Proto-Calepineae Karyotype, ancestral genome of *Brassica* species, *CCB* conserved collinear block, *LF* the least fractionated blocks of *Brassica*, *MF1* the medium fractionated blocks of *Brassica*, *MF2* the most fractionated blocks of *Brassica*, *PL* protein length, *MW* molecular weight.

The physical and chemical characteristics of the SWEET proteins in *B. oleracea* were predicted, and their sizes were found to range from 147 aa (BoSWEET7b) to 475 aa (BoSWEET5c), with the corresponding open reading frames (ORF) ranging from 441 bp to 1425 bp. The predicted molecular weights ranged from 16.20 kDa (BoSWEET7b) to 53.51 kDa (BoSWEET5c) (Table 1), and the theoretical isoelectric points (pI) from 6.28 (BoSWEET15b) to 9.56 (BoSWEET7a), with most, except for three, being higher than 7.60 (Table 1).

### Chromosomal distribution and differential retention of *SWEET* genes in *B. oleracea*

**Fig. 3:**
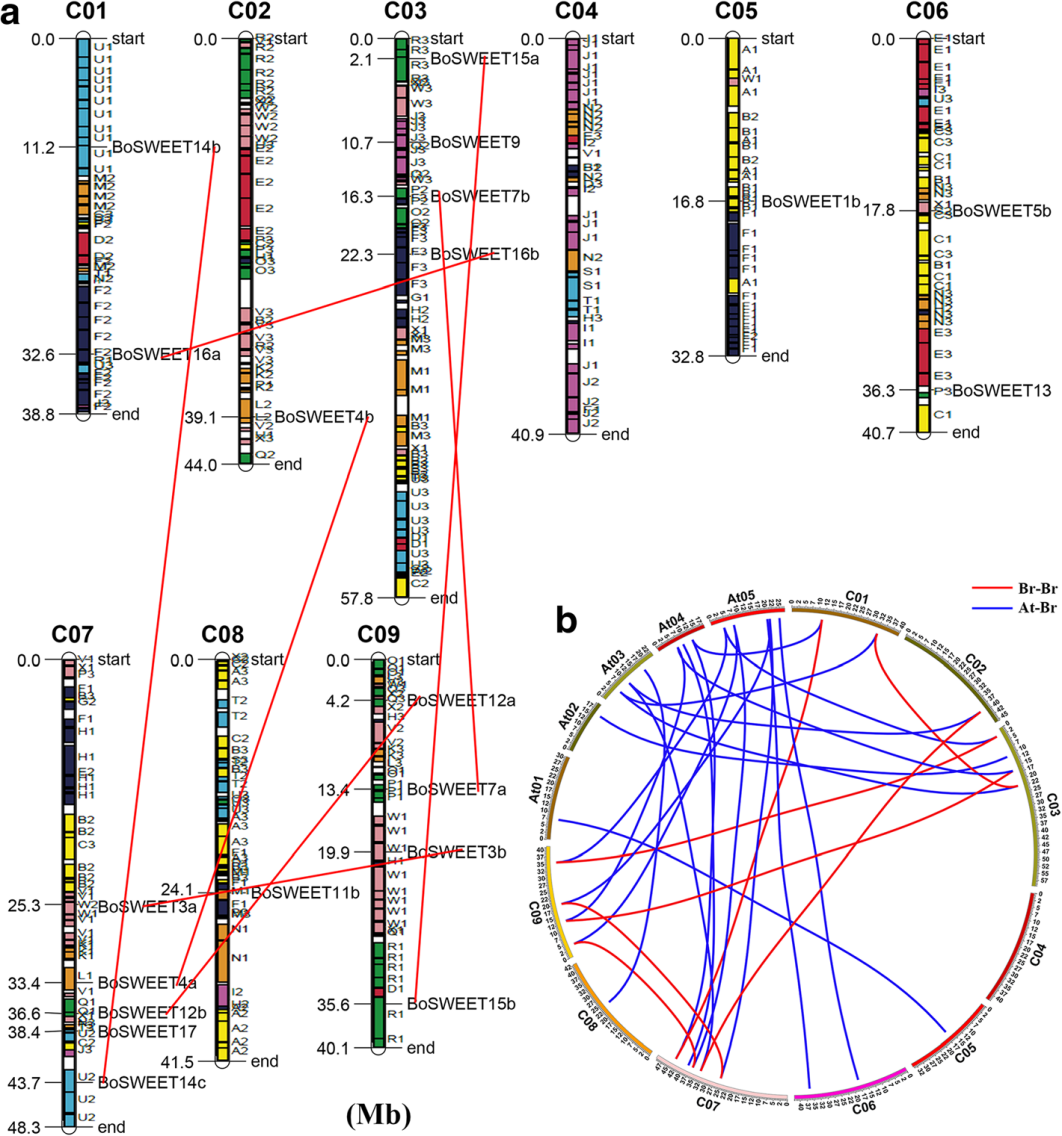
Distribution of *BoSWEET* genes on the nine cabbage chromosomes and the retention of *SWEET* genes between *B. oleracea* and *A. thaliana*. **a** Distribution of *BoSWEET* genes. The 24 conserved collinear blocks on each chromosome are labeled A–X and three subgenomes are plotted, based on a previous report [44]. The *BoSWEET* genes present on duplicated chromosomal segments are shown connected by red lines between the two relevant chromosomes. **b** Syntenic relationship of *B. oleracea* and *A. thaliana SWEET* genes shown on the chromosome maps

The three subgenomes of *B. oleracea* (LF, the least fractionated blocks of *Brassica*; MF1, the most fractionated blocks of *Brassica*; MF2, the medium fractionated blocks of *Brassica*) have been established to distinguish the degree of fractionation in genome evolution [44]. In this study, 29 *BoSWEET* genes (i.e., the whole family except *BoSWEET13*) were distributed among the three subgenomes (11 *BoSWEET* genes in LF, 9 in MF1, and 9 in MF2) (Table 1). Interestingly, all the *BoSWEET* genes were retained in *B. oleracea* after triplication and fractionation, except for the loss of *BoSWEET6*. Approximately half of the *BoSWEET* genes (8/17) were retained in two copies, while only five (*BoSWEET8*, *− 9*, *− 10*, *− 13* and *− 17*) and three (*BoSWEET5*, *BoSWEET11* and *BoSWEET14*) were retained in one and three copies, respectively (Table 1). The retained copies have the same conserved collinear block. The physical positions of the *BoSWEET* genes on the *B. oleracea* chromosomes were identified (Fig. 3a), which revealed that 20 (66.7%) were distributed across the nine chromosomes (C01-C09), with the exception of the C04 chromosome having no members, while the largest number on any single chromosome was five on chromosome 7 (Fig. 3a). Ten *SWEET* genes (*BoSWEET1a, −2a, −2b, −5a, −5c, − 8, − 10, −11a, −11c* and *-14a*) were not anchored on any of the *B. oleracea* chromosomes.

In eukaryotic genomes, a substantial proportion of protein-coding genes belong to multigene families, which have presumably evolved by the process of gene duplication. Duplicated genes from eukaryotic genomes have originated predominantly from inter-chromosomal duplications [69]. We observed that seven *BoSWEET* genes underwent segment duplication (7 duplications), leading to many homologs on different chromosomes, as indicated with red lines in Fig. 3a. To further highlight the specific retention of *BoSWEET* genes, their syntenic relationship with *AtSWEET* genes was determined using the Circos program (Fig. 3b).

### *Ks* analysis of *B. oleracea* and *A. thalianaSWEET* genes

**Fig. 4:**
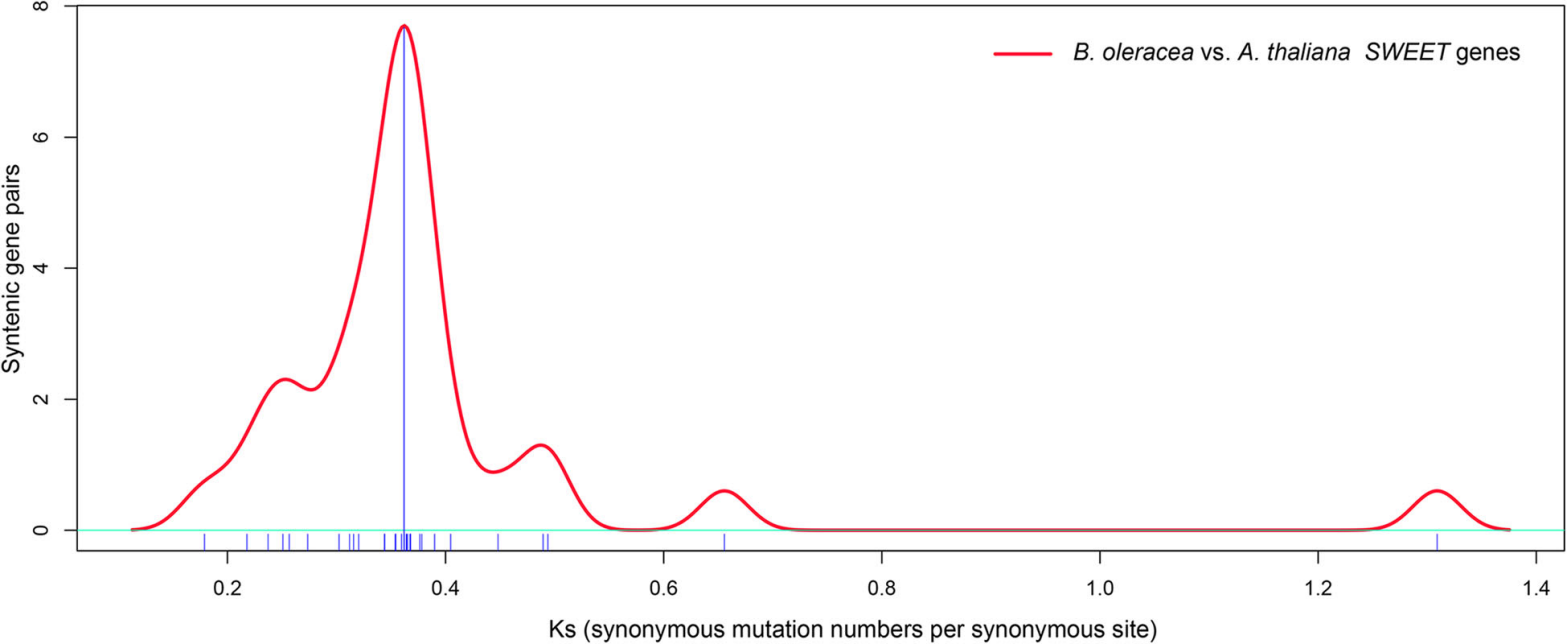
Distribution of *Ks* values of *SWEET* orthologous gene pairs between *B. oleracea* and *A. thaliana*

The *Ks* and *Ka* values of 30 syntenic *SWEET* orthologous pairs between *B. oleracea* and *A. thaliana* were analyzed (Table 2, Additional file 4: Table S3). The *Ka*/*Ks* ratios of all the *SWEET* syntenic orthologous pairs were far less than 1, indicative of purifying selection for retention. To estimate the divergence time between the two species, the *Ks* values of the orthologous *SWEET* genes were used and observed to range from 0.3 to 0.45, concentrating at approximately 0.35 (Fig. 4). According to the neutral substitution rate of 1.5 × 10^− 8^ substitutions per site per year for dicotyledonous plants [60], this suggests the *SWEET* gene family of *B. oleracea* diverged from *A. thaliana* approximately 10 to 15 million years ago (MYA). We concluded that the *SWEET* genes diverged concurrently with the Brassica-specific WGT event that occurred approximately 13–17 MYA [45, 70].

**Table 2:** Non-synonymous (*Ka*) and synonymous substitution rate (*Ks*) between orthologous *SWEET* gene pairs in *B. oleracea* and *A. thaliana*

Orthologous gene pairs		*Ka*	*Ks*	*Ka*/*Ks*	Duplication date (MYA)
*AtSWEET1*	*BoSWEET1a*	0.0393	0.4481	0.0877	14.9
	*BoSWEET1b*	0.0404	0.3674	0.1100	12.2
*AtSWEET2*	*BoSWEET2a*	0.0458	0.2509	0.1825	8.4
	*BoSWEET2b*	0.0756	0.368	0.2054	12.3
*AtSWEET3*	*BoSWEET3a*	0.1024	0.3765	0.2720	12.6
	*BoSWEET3b*	0.1163	0.39	0.2982	13.0
*AtSWEET4*	*BoSWEET4a*	0.0491	0.3639	0.1349	12.1
	*BoSWEET4b*	0.0624	0.3623	0.1722	12.1
*AtSWEET5*	*BoSWEET5a*	0.0709	0.3544	0.2001	11.8
	*BoSWEET5b*	0.0509	0.3204	0.1589	10.7
	*BoSWEET5c*	0.0853	0.3782	0.2255	12.6
*AtSWEET7*	*BoSWEET7a*	0.0863	0.4894	0.1763	16.3
	*BoSWEET7b*	0.1344	0.6556	0.2050	21.9
*AtSWEET8*	*BoSWEET8*	0.2009	1.3091	0.1535	43.6
*AtSWEET9*	*BoSWEET9*	0.0642	0.2567	0.2501	8.6
*AtSWEET10*	*BoSWEET10*	0.101	0.4938	0.2045	16.5
*AtSWEET11*	*BoSWEET11a*	0.0434	0.2373	0.1829	7.9
	*BoSWEET11b*	0.0214	0.2179	0.0982	7.3
	*BoSWEET11c*	0.0287	0.179	0.1603	6.0
*AtSWEET12*	*BoSWEET12a*	0.0599	0.3024	0.1981	10.1
	*BoSWEET12b*	0.0476	0.3442	0.1383	11.5
*AtSWEET13*	*BoSWEET13*	0.0961	0.2736	0.3512	9.1
*AtSWEET14*	*BoSWEET14a*	0.0712	0.312	0.2282	10.4
	*BoSWEET14b*	0.0652	0.3597	0.1813	12.0
	*BoSWEET14c*	0.0967	0.3643	0.2654	12.1
*AtSWEET15*	*BoSWEET15a*	0.1207	0.3539	0.3411	11.8
	*BoSWEET15b*	0.1114	0.344	0.3238	11.5
*AtSWEET16*	*BoSWEET16a*	0.0912	0.3653	0.2497	12.2
	*BoSWEET16b*	0.1011	0.4047	0.2498	13.5
*AtSWEET17*	*BoSWEET17*	0.0457	0.3157	0.1448	10.5

### *BoSWEET* gene structures and associated conserved protein motifs

**Fig. 5:**
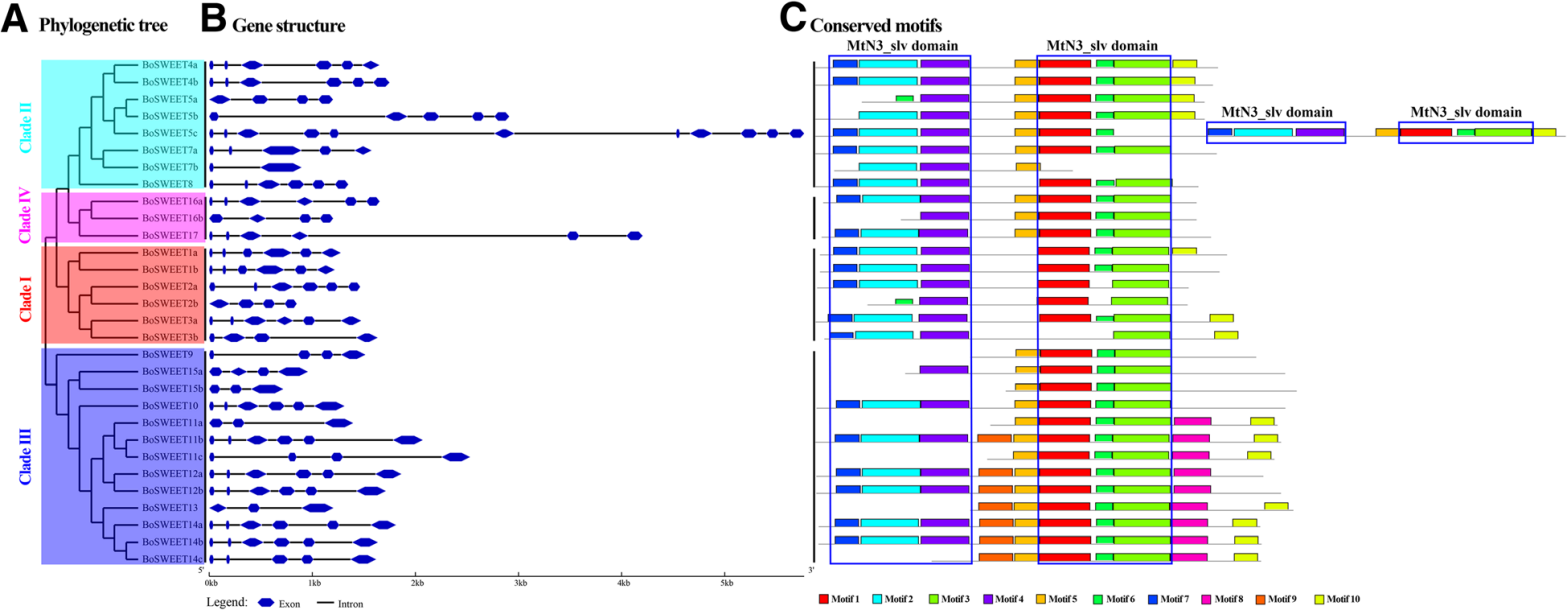
*BoSWEET* gene and protein structures. **a** Unrooted phylogenetic tree based on the full-length amino acid alignment of all the BoSWEET proteins. The phylogenetic tree was constructed using the neighbor-joining (NJ) method and with 1000 bootstrap replications. *BoSWEET* genes were grouped according to their phylogenetic classification. **b** Structures of *BoSWEET* genes**.** Exons and introns are represented by blue double-sided wedges and black lines, respectively. **c** Distribution of conserved motifs in the *BoSWEET* proteins. Different motifs are indicated by different colors and numbered 1–7. Same number in different proteins refers to the same motif. Motifs 7, 2, 4 and motifs 1, 6, 3 were annotated as MtN3_slv domains, respectively

Gene structural diversity and conserved protein motif divergence played key roles in the evolution of the *SWEET* gene family [71]. The exon-intron organization of the cabbage *SWEET* genes was analyzed, and half (15 members) were found to have six exons, while seven members had four exons, three had five exons, three had three exons and *BoSWEET7b* and *BoSWEET5c* had 2 and 11 exons (Fig. 5), respectively. The exon lengths were similar, while the intron length varied, with eight *BoSWEETs* (*BoSWEET3b*, *−5b*, *− 5c*, *− 9*, *−11a*, *−11b*, *−11c* and *− 17*) containing very long introns (Fig. 5). Six genes appeared to have exon-intron loss variations. For example, *BoSWEET2b* (four exons) lost the first two exons and two introns compared with *BoSWEET2a*; *BoSWEET5a* (four exons) lost the first exon and intron compared with *BoSWEET5b*; *BoSWEET11a* (three exons) lost the first three exons and introns compared with *BoSWEET11b*; and *BoSWEET14c* lost the third exon compared with *BoSWEET14b* (Additional file 5: Figure S2).

Among the 30 BoSWEET proteins, we found that the most BoSWEET (21) contained two MtN3_slv domains, a characteristic eukaryote SWEET protein domain, whereas eight contained only one MtN3_slv domain (Table 1). The number of TMHs ranged from three to eleven and concentrated in seven (Additional file 6: Figure S3). However, we identified the BoSWEET5c protein with four MtN3_slv domains that has a similar predicted protein architecture to VV14G09070 from *V. vinifera*, and that together constitute a novel sub-type, which were named extra-SWEET (Additional file 7: Figure S4). To further investigate the structural diversity, the conserved motif structure of all the BoSWEET proteins was analyzed. In total, ten conserved motifs (motifs 1–10) were identified, among which motifs 7, 2, 4, and motifs 1, 6, 3 were annotated as MtN3_slv domains, respectively. Motif 5 or motifs 9, 5 connects the two MtN3_slv domains (Fig. 5). In addition, none of the 30 BoSWEET proteins were predicted to contain signal peptide sequences.

### Characterization of *BoSWEET* expression in response to chilling stress

**Fig. 6:**
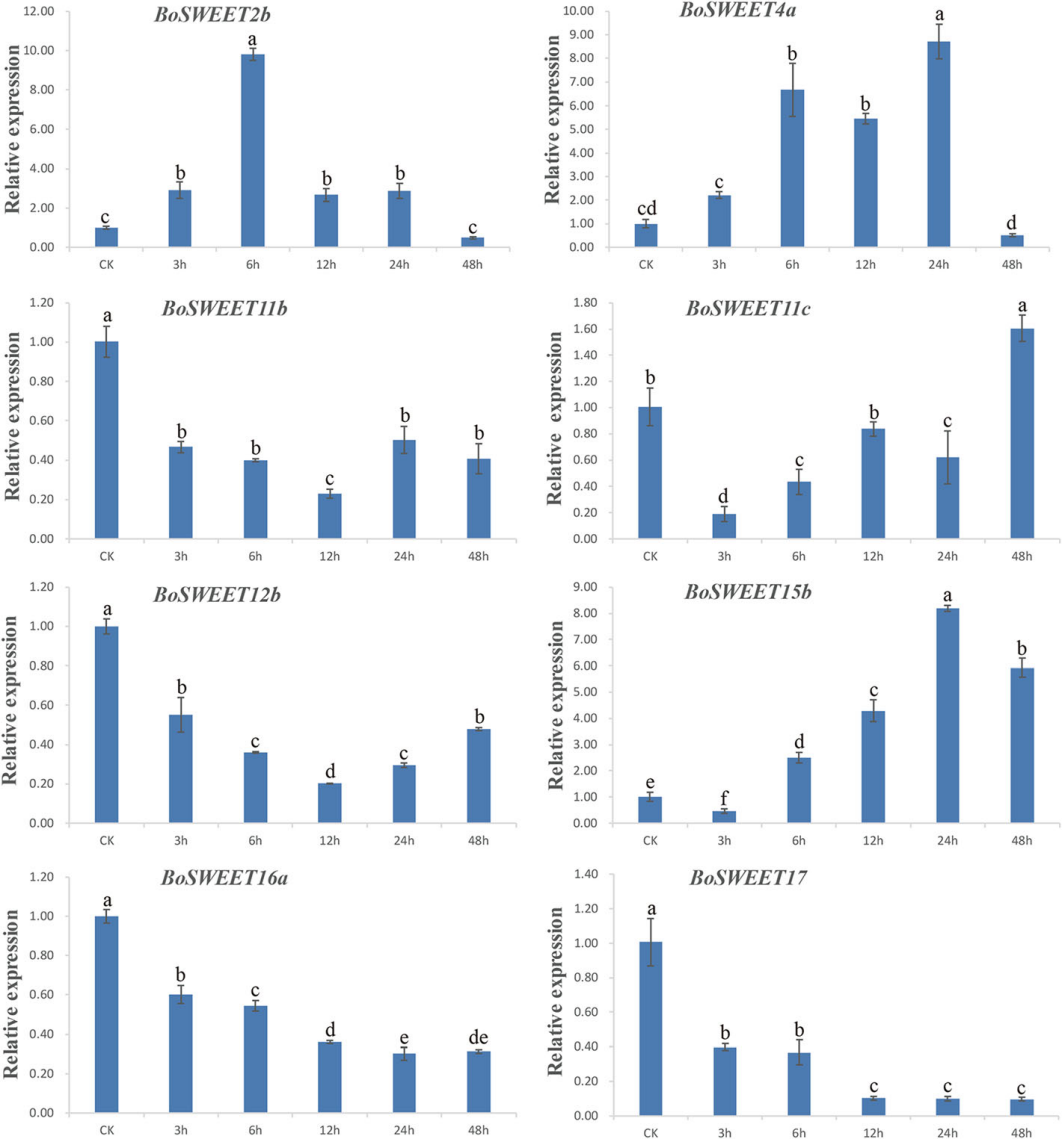
qRT-PCR analysis of eight *BoSWEET* genes in cabbage leaves following chilling treatment. Data are presented as means ± standard deviations of three technical replicates derived from one bulked biological replicate. A Duncan’s multiple range test was used to calculate the significance level of the data at *P* < 0.05

A total of eight *BoSWEET* genes were selected for expression pattern analysis using the chilling-tolerant line CT-923. The eight selected *BoSWEET* genes represented all four clades, with *BoSWEET2b* and *BoSWEET4a* belonging to clade I and clade II, respectively; *BoSWEET11b, BoSWEET11c, BoSWEET12b* and *BoSWEET15b* belonging to clade III, and *BoSWEET16a* and *BoSWEET17* belonging to clade IV. We found that expression of *BoSWEET2b*, *BoSWEET4a* and *BoSWEET15b* were induced after chilling stress (Fig. 6), with *BoSWEET2b* expression increasing to maximal levels at 6 h following chilling stress. *BoSWEET4a* and *BoSWEET15b* expression peaked at 24 h after chilling stress. In addition, while the expression of *BoSWEET11b, BoSWEET11c, BoSWEET12b*, *BoSWEET16a* and *BoSWEET17* decreased in response to chilling, the expression patterns were different. The expression of *BoSWEET11b* and *BoSWEET12b* declined to a minimum at 12 h, then rose to reach nearly half of the expression of the control. The expression of *BoSWEET11c* declined sharply to a minimum at 3 h after treatment, then increased to a maximum at 48 h. *BoSWEET16a* and *BoSWEET17* had a similar expression pattern after chilling exposure, with expression declining rapidly and remaining at a low level even after 12 h (Fig. 6).

### Expression of *BoSWEET* genes in different organs and in response to *P. brassicae* infection

**Fig. 7:**
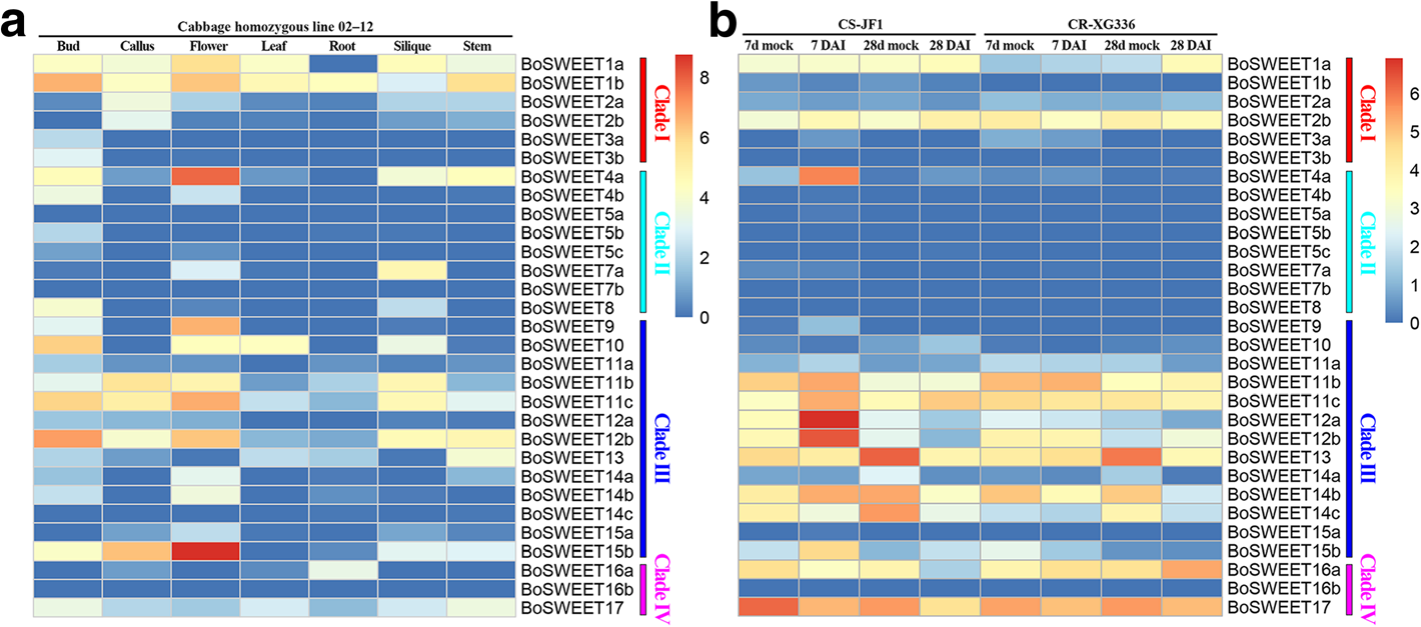
Expression patterns of *BoSWEET* genes analyzed by RNA-Seq. **a** Heatmap showing *BoSWEET* gene expression in different organs. Bud, callus, flower, leaf, root, silique and stem are represented. **b** Heatmap showing expression dynamics of *BoSWEET* genes in cabbage roots following *P. brassicae* infection. Expression levels of the *BoSWEET* genes are shown as the Log2 transformed FPKM values obtained from the RNA-Seq data. DAI, days after inoculation

We examined the RNA-Seq data set (GSE42891) to determine transcript levels of the *BoSWEET* genes in the cabbage bud, callus, flower, leaf, root, silique and stem. The expression of most of the *BoSWEET* genes exhibited different patterns (Fig. 7a). Seven were expressed in all organs, whereas the expression of three was not detected. Several genes were expressed in only one or two organ types, such as *BoSWEET3a* in buds, *BoSWEET3b* and *BoSWEET5c* in buds and flowers, *BoSWEET14c* in roots and stems, and *BoSWEET16a* in calli and roots (Fig. 7a). This diversity of expression patterns suggested a broad range of biological functions of *BoSWEET* genes during growth and development of cabbage.

We also examined the expression patterns of *BoSWEET* genes in CR-XG336, a clubroot-resistant line, and CS-JF1, a clubroot-susceptible line, infected by *P. brassicae* at two infection stages, and observed variation between the two different cultivars and at different infection stages (Fig. 7b). Several genes were nearly undetectable in both cultivars both before *P. brassicae* infection and at two infection stages. Six (*BoSWEET4a*, *−11c*, *−12a*, *−12b*, *−14b and -15b*) and three (*BoSWEET14c*, *BoSWEET16a* and *BoSWEET17*) *BoSWEET* genes were significantly up- and down-regulated, respectively, in CS-JF1 at 7 DAI (primary infection stage) compared with mock-treated plants. No *BoSWEET* genes were significantly up- or down-regulated in CR-XG336 at 7 DAI compared with mock-treated plants (Fig. 7b, Additional file 8: Table S4). Moreover, at 28 DAI (clubroot formation stage), six *BoSWEET* genes (*BoSWEET12b*, *− 13*, *−14a*, *−14b*, *−14c* and *− 17*) were significantly down-regulated in CS-JF1 after infection, whereas two (*BoSWEET1a* and *BoSWEET16a*) and three (*BoSWEET13*, *BoSWEET14a* and *BoSWEET14b*) were up- and down-regulated in CR-XG336, respectively (Fig. 7b, Additional file 8: Table S4).

### Subcellular localization analysis of BoSWEET proteins following heterologous expression in tobacco

**Fig. 8:**
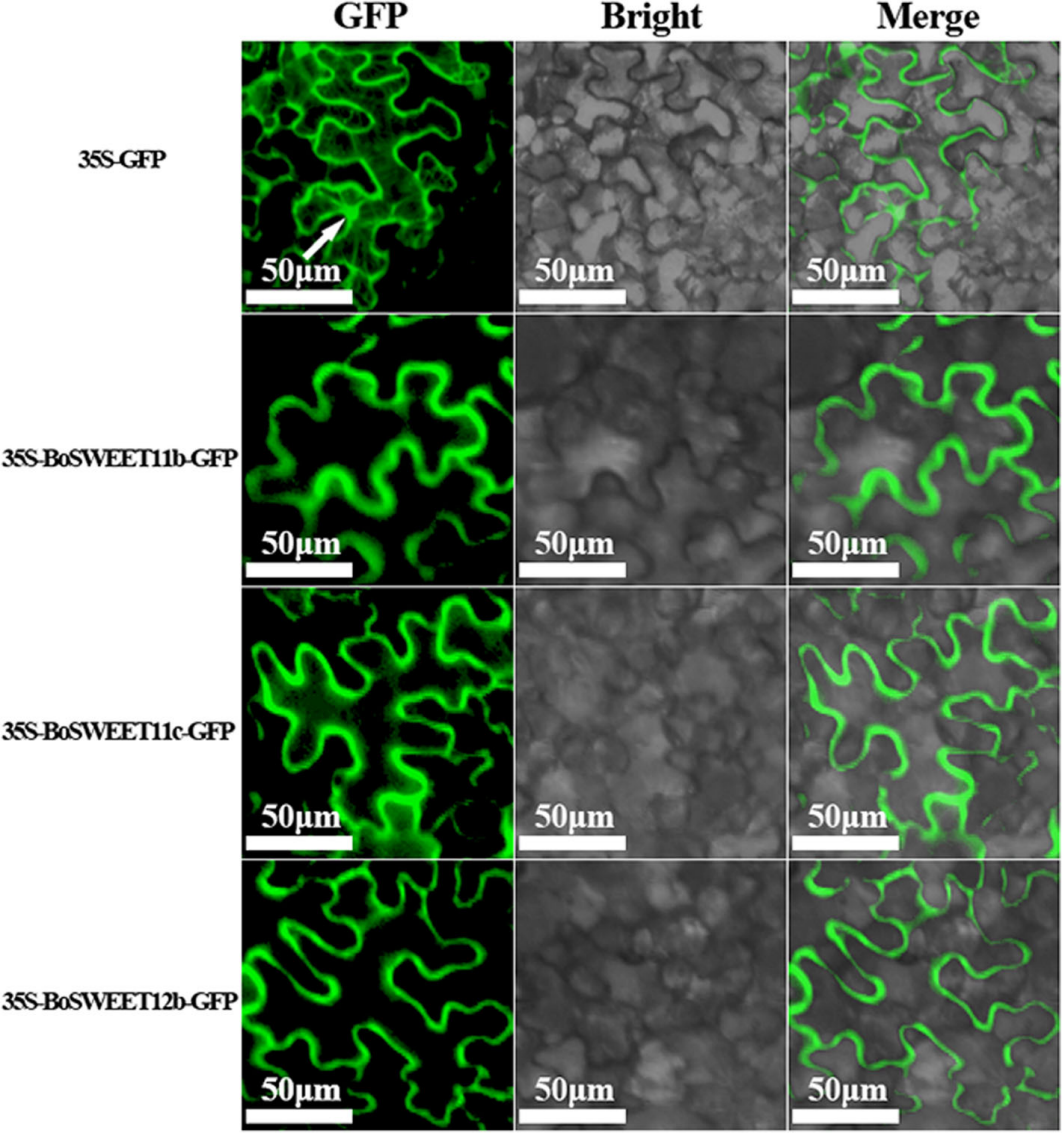
Subcellular localization of BoSWEET proteins in *N. benthamiana* leaves. BoSWEET11b-GFP, BoSWEET11c-GFP, BoSWEET12b-GFP fusion proteins, as well as GFP alone, were transiently expressed in *N. benthamiana* leaves using *Agrobacterium* infiltration. Protein localization was examined 48 h after infiltration and representative images are shown

To study the subcellular localization of BoSWEET proteins, BoSWEET11b, BoSWEET11c and BoSWEET12b were heterologously and transiently expressed in tobacco leaf epidermal cells as translational GFP fusion proteins. All three proteins were found to be localized to the plasma membrane (PM), whereas the control 35S-GFP (GFP alone) was detected in the PM, cytoplasm and nucleus (Fig. 8). These results suggested that all three proteins are PM-localized (Fig. 5, Additional file 5: Figure S2).

## Discussion

### Evolutionary history of *SWEET* genes among fourteen plant species

Gene family evolution is characterized by gene duplication via whole-genome duplication (WGD), tandem gene duplication and segmental duplication events [72]. Following duplication, duplicated gene pairs can undergo different fates, including neo-functionalization (functional diversification), sub-functionalization (partitioning of the function between daughter copies) and non-functionalization [73, 74]. In plants, genome duplication has been shown to contributed their ability to adapt to diverse environments, including drought, pathogen attack, and extreme temperatures, as well as reproductive development [75]. In this study, the number of *SWEET* genes was found to vary considerably among fourteen plant species. For example, there were only 1–3 members in algae, with the salt water species *O. lucimarius* and *O. tauri* having only one copy and the fresh water species *V. carteri* and *C. reinhardtii* having three. The evolution from unicellular (algae) to multicellular plants led to further expansion of the *SWEET* gene family. The bryophyte *P. patens* is an early diverging land plant with only one primitive protophloem and has six *SWEET* gene family members, while the pteridophyta *S. moellendorffii* evolved to have phloem and has 15 *SWEET* genes, which is similar to the number in many angiosperms (Fig. 2). These results suggested that the expansion of *SWEET* genes might play an important role in land plant adaptations to terrestrial conditions.

Among Brassicaceae species, *B. oleracea*, *B. rapa* and *A. thaliana* have experienced γ, β and α WGD events. The number of *SWEET* genes in *B. oleracea* (30) and *B. rapa* (33) nearly doubled when compared with the number in *A. thaliana* (17) after the *Brassica*-specific WGT event, during which genes had experienced triplication, differentiation and fractionation (loss) [45, 70]. However, the gene balance hypothesis assumes that genes participate in macromolecular complexes, signaling and transcriptional networks are preferentially retained, thus avoiding the instability and unfitness of network caused by the imbalance associated with loss of one member of a complex [76–78]. Similarly, we found evidence of *SWEET* gene fractionation in *B. oleracea* after the split with *A. thaliana* from the recent common ancestor, with *BoSWEET6* being lost, five *BoSWEETs* (*BoSWEET8*, *− 9*, *− 10*, *− 13* and *− 17*) and eight *BoSWEETs* (*BoSWEET1*, *− 2*, *− 3*, *− 4*, *− 7*, *− 12*, *− 15*, *− 16*) having one and two separate orthologous genes in *A. thaliana*, respectively (Table 1; Additional file 3: Figure S1). WGD or WGT generates the multiplicity in gene copy numbers, and is the main source of genetic redundancy. We firmly believe that changes in *SWEET* genes during the WGT event played critical roles in the adaptation and expansion of rich morphotypes of *Brassica* crops.

### Structure and evolution of *BoSWEET* genes

The SWEET proteins have evolved from an internal repeat duplication of three-TMH unit and fusion with an insertion of TMH4 [8]. In this study, we searched for ten conserved motifs among the 30 BoSWEET proteins and found the MtN3_slv domains were located in duplication regions in almost all BoSWEET proteins (Fig. 5). The extra-SWEET protein (BoSWEET5c, 11-TMHs) may have originated from two internal SWEET (7-TMHs, two MtN3_slv domains) duplications, similar to the duplication of semi-SWEET (3-TMHs, one MtN3_slv domain) and subsequently evolved into the SWEET [8, 79]. In *V. vinifera*, berries accumulate high levels of sugars, and the SWEET protein, VV14G09070, might play a role in mediating elevated levels of sucrose transport. To this end, it would be very interesting to investigate the spatial expression pattern of *VV14G09070* and its potential function in long distance sugar transport during flower or berry development [80].

### Expression patterns of *BoSWEET* genes in different organs and in response to chilling stress

Transcriptome sequencing revealed that more *BoSWEET* genes were highly expressed in flowers and buds than in the other organs investigated. Of the highly expressed *BoSWEET* genes, most were in clade III (Fig. 7a), which is consistent with studies of rapeseed (*Brassica napus*) [20]. The expression of *SWEET* genes has been shown to change in response to abiotic stress in several plant species [19, 26, 81]. For example, in tomato *SlSWEET10a*, −*10b*, −*10c*, −*11a*, −*11b*, −*11c*, −*11d*, −*12a* and -*12c* were reported to have similar expression patterns and were up-regulated several fold in leaves but substantially down-regulated in roots in response to sugar treatment, and salt, heat and cold stresses [19]. We observed that the expression of *BoSWEET11b*, *−11c*, *−12b*, *−16a* and *− 17* was down-regulated by chilling stress (Fig. 6), which may contribute to the accumulation of glucose and fructose in leaves, leading to increased chilling tolerance. Heterologously expressed BoSWEET11b, BoSWEET11c and BoSWEET12b proteins fused to a GFP marker were observed to be accumulated in the PM, consistent with a role in mediating sucrose efflux from phloem parenchyma cells into the sieve element-companion cell complex [9]. The expression of *BoSWEET16a* and *BoSWEET17* declined rapidly after chilling stress and remained at low levels from 12 h to 48 h. The orthologous genes from *A. thaliana*, *AtSWEET16* and *AtSWEET17*, have been reported to export fructose from the vacuole and contribute to cytosolic fructose homeostasis [11, 12, 26].

### Involvement of *BoSWEET* genes in the response to *P. brassicae* infection

Previous studies revealed that *P. brassicae* obtains sugars from hosts to complete its life cycle, involving the formation of galls, which act as an additional sink. In *A. thaliana,* it was found that sucrose accumulated in uninfected leaves, but not in *P. brassicae* infected leaves because sucrose was exported from leaves into the clubroot galls [82]. The expression of sugar transporter genes may therefore influence plant-*P. brassicae* interactions. In this study, the expression of six *BoSWEET* genes (*BoSWEET4a*, −*11c*, −*12a*, −*12b*, −*14b* and -*15b*) was up-regulated in roots in the susceptible CS-JF1 upon *P. brassicae* infection at 7 DAI compared with mock plants. In contrast, no *BoSWEET* genes were up-regulated in the resistant CR-XG336 at 7 DAI (Fig. 6; Additional file 8: Table S4). We infer from these results that these six *BoSWEET* genes could be responsible for transporting sugars to the sink roots associated with *P. brassicae* colonization in CS-JF1. Clade III SWEET proteins have been shown to function as sucrose transporters involved in long distance sugar transport in *A. thaliana* [3, 9], consistent with our observation that all the up-regulated *BoSWEET* genes involved in *P. brassicae* infection in CS-JF1, except *BoSWEET4a*, belong to clade III. Clade III SWEET proteins have also been shown to be involved in sugar transport in clubroot disease establishment [30]. We also noted that the expression of the *BoSWEET1a* and *BoSWEET16a* genes was approximately 4-fold and 2-fold higher, respectively, in CR-XG336 at 28 DAI than in the uninfected control (Additional file 8: Table S4). Whether the increased expressions of the two *BoSWEET* genes contribute to CR-XG336 resistance to *P. brassicae* infection stress remains to be established.

## Conclusions

In this study, 30 *BoSWEET* genes were identified in the *B. oleracea* genome and further clustered into four clades based on a phylogenetic tree of 205 *SWEET* homologs from fourteen representative plant species. Clade II was evolutionarily the most ancient, while clade I, clade IV and clade III were formed successively. The ORF lengths of the *BoSWEET* genes ranged from 441 bp to 1425 bp, and the *Ks* values of the orthologous *SWEET* genes from *B. oleracea* and *A. thaliana* ranged from 0.30 to 0.45, meaning that the estimated time of divergence of the two species was approximately 10 to 15 MYA. Eight BoSWEET proteins were predicted to contain a single MtN3_slv domain, twenty-one to contain two, and one (BoSWEET5c) to have four. qRT-PCR analysis showed that the expression of five *BoSWEET* genes decreased when plants were exposed to chilling stress. The expression of six *BoSWEET* genes was up-regulated in CS-JF1 following *P. brassicae* infection at 7 DAI compared with mock controls, and we hypothesize that they might be responsible for transporting sugars to the sink roots during *P. brassicae* colonization. Overall, these findings facilitate unraveling the potential candidate *BoSWEET* genes involved in the response to chilling and clubroot disease, and provide valuable information to facilitate the breeding of chilling tolerant and clubroot disease-resistant cultivars in cabbage.

## Additional files

Additional file 1: **Table S1.** Primers used in this study. (XLSX 10 kb)

Additional file 2: **Table S2.** Amino acid sequences of 205 SWEET proteins from 14 plant species. (XLSX 35 kb)

Additional file 3: **Figure S1.** Phylogenetic tree of *B. oleracea*, *B. rapa* and *A. thaliana* SWEET proteins. Phylogenetic analysis of 80 SWEET proteins from *B. oleracea* (30), *B. rapa* (33) and *A. thaliana* (17) showing similar groups in all species. Four clades were marked with different colors. (TIF 1595 kb)

Additional file 4: **Table S3.** Nucleotide sequences of 47 *SWEET* genes from *B. oleracea* and *A. thaliana*. (XLSX 19 kb)

Additional file 5: **Figure S2.** Gene organization of *BoSWEET* genes. (TIF 290 kb)

Additional file 6: **Figure S3.** Protein structure of BoSWEETs in *B. oleracea*. Red rectangles signify the TMHs, and blue and carmine lines indicate the intracellular and extracellular regions, respectively. (TIF 545 kb)

Additional file 7: **Figure S4.** Conserved domain architecture of the BoSWEET5c and VV14G09070 proteins. (TIF 177 kb)

Additional file 8: **Table S4.** Expression of *BoSWEET* genes in roots of clubroot-resistant and susceptible cabbage lines at different *P. brassicae* infection stages. (XLSX 18 kb)

## Abbreviations

CDART: Conserved Domain Architecture Retrieval Tool
GSDS: Gene Structure Display Server
HMM: Hidden Markov model
*Ka*: Nonsynonymous substitution rate
*Ks*: Synonymous substitution rate
MEME: Multiple EM for Motif Elicitation
qRT-PCR: Quantitative reverse transcription-PCR
SMART: Simple Modular Architecture Research Tool
TMH: Transmembrane helix
WGD: Whole-genome duplication
WGT: Whole-genome triplication
