# Supplementary figures and images for: Genome-wide characterization and expression profiling of SWEET genes in cabbage (Brassica oleracea var. capitata L.) reveal their roles in chilling and clubroot disease responses
Source: BMC Genomics. 2019 Jan 29;20:93. doi: 10.1186/s12864-019-5454-2 (PMC6352454; doi:10.1186/s12864-019-5454-2)

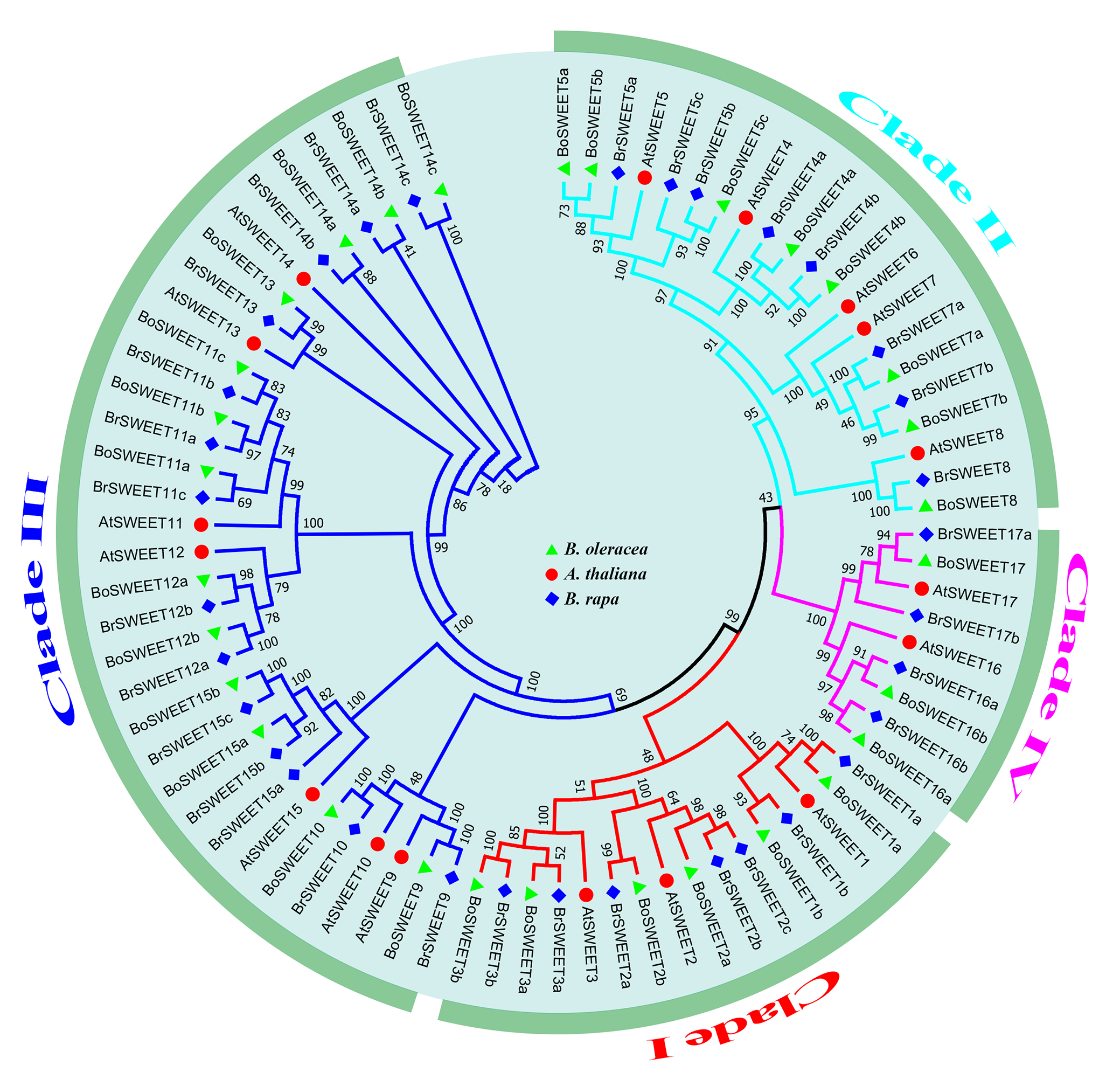

Supplement: Supplementary file 3 — Figure S1. Phylogenetic tree of B. oleracea, B. rapa and A. thaliana SWEET proteins. Phylogenetic analysis of 80 SWEET proteins from B. oleracea (30), B. rapa (33) and A. thaliana (17) showing similar groups in all species. Four clades were marked with different colors. (TIF 1595 kb) [file 12864_2019_5454_MOESM3_ESM.tif]

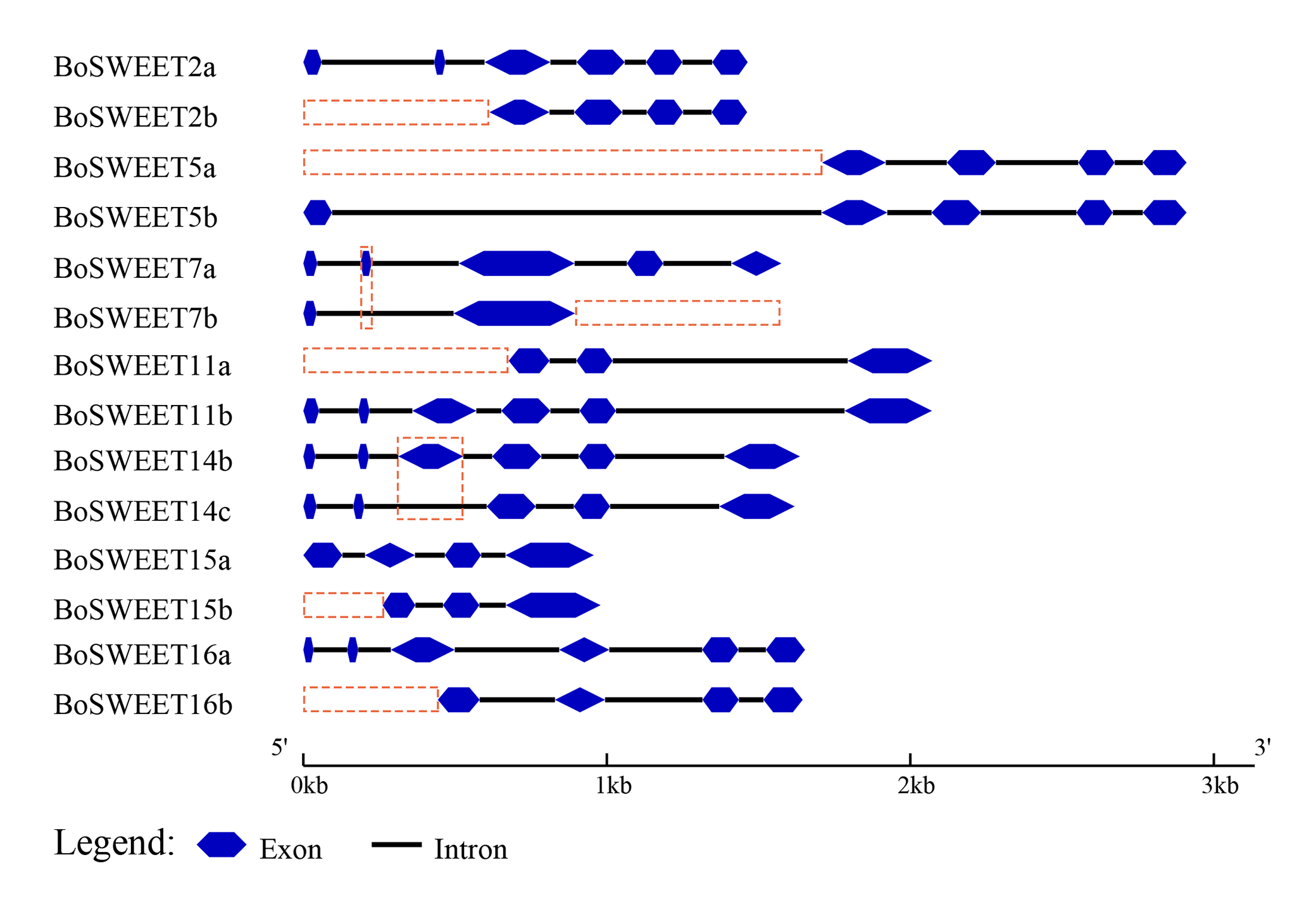

Supplement: Supplementary file 5 — Figure S2. Gene organization of BoSWEET genes. (TIF 290 kb) [file 12864_2019_5454_MOESM5_ESM.tif]

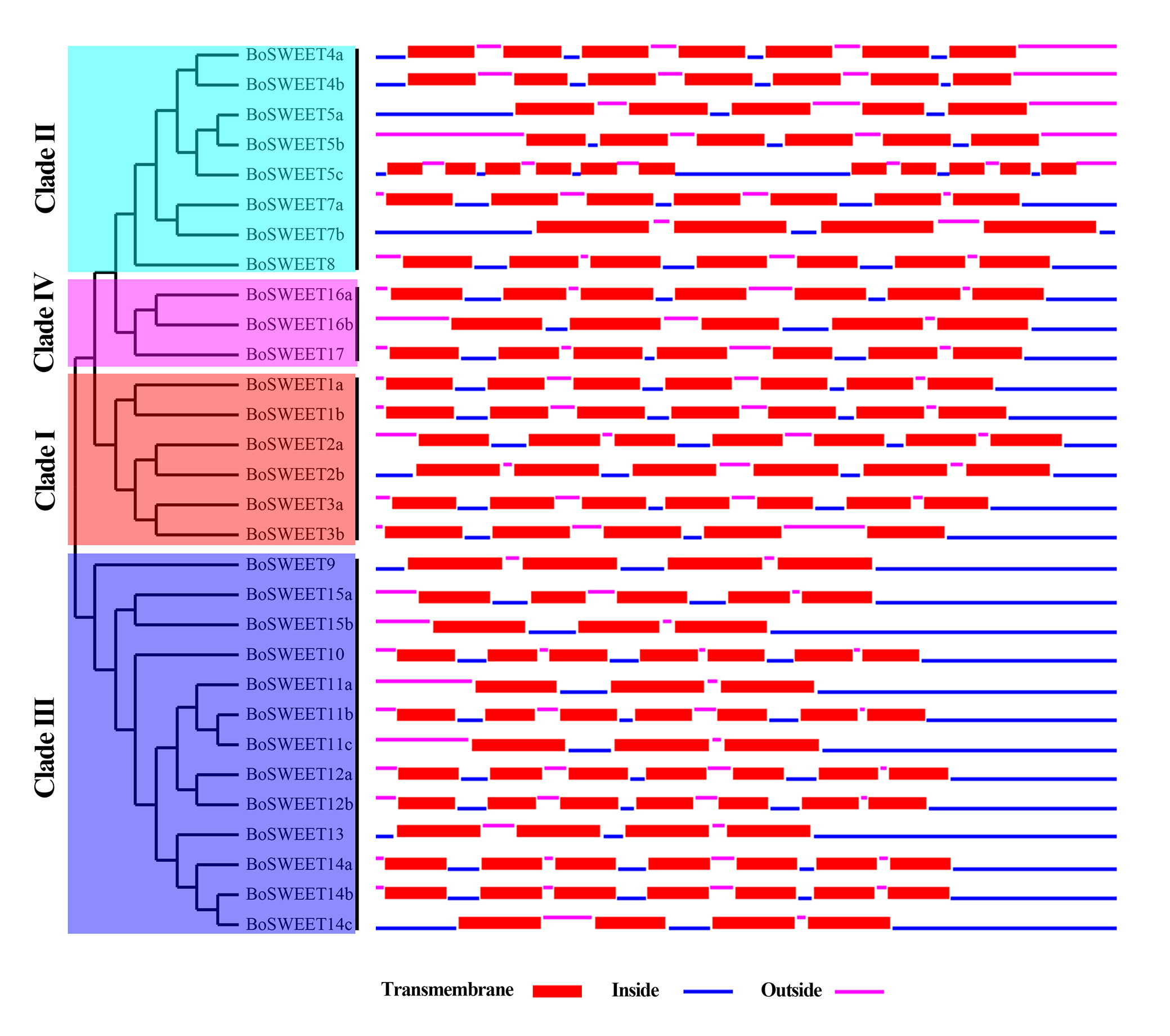

Supplement: Supplementary file 6 — Figure S3. Protein structure of BoSWEETs in B. oleracea. Red rectangles signify the TMHs, and blue and carmine lines indicate the intracellular and extracellular regions, respectively. (TIF 545 kb) [file 12864_2019_5454_MOESM6_ESM.tif]

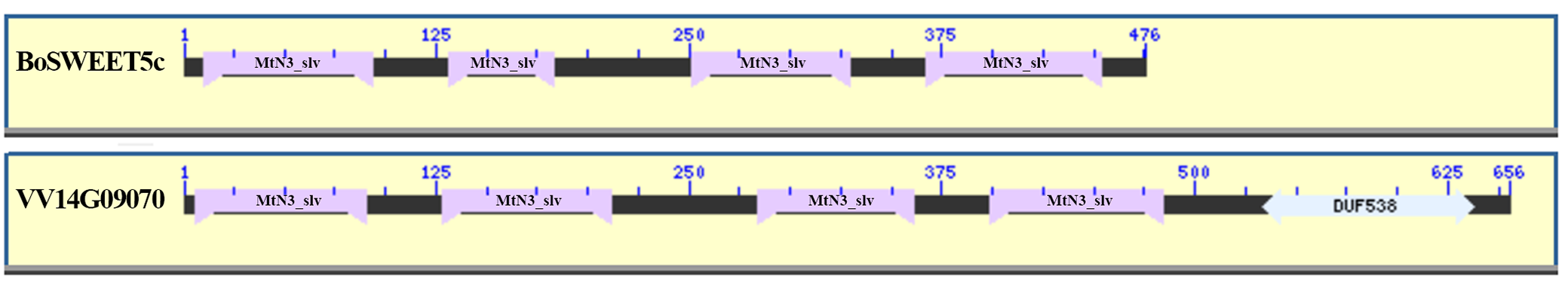

Supplement: Supplementary file 7 — Figure S4. Conserved domain architecture of the BoSWEET5c and VV14G09070 proteins. (TIF 177 kb) [file 12864_2019_5454_MOESM7_ESM.tif]
